# The Improvement of the Resistance to *Candida albicans* and *Trichophyton interdigitale* of Some Woven Fabrics Based on Cotton

**DOI:** 10.1155/2014/763269

**Published:** 2014-09-04

**Authors:** Lilioara Surdu, Maria Daniela Stelescu, Elena Manaila, Gheorghe Nicula, Ovidiu Iordache, Laurentiu Christian Dinca, Mariana-Daniela Berechet, Mariana Vamesu, Dana Gurau

**Affiliations:** ^1^National R&D Institute for Textile and Leather, 16 Lucretiu Patrascanu Street, 030508 Bucharest, Romania; ^2^National R&D Institute for Textile and Leather, Leather and Footwear Research Institute, 93 Ion Minulescu Street, 031215 Bucharest, Romania; ^3^National Institute for Laser, Plasma and Radiation Physics, Electron Accelerators Laboratory, 409 Atomistilor Street, 077125 Magurele, Romania

## Abstract

This paper presents the improvement of the antimicrobial character of woven fabrics based on cotton. The woven fabrics were cleaned in oxygen plasma and treated by padding with silver chloride and titanium dioxide particles. The existence of silver and titanium on woven fabrics was evidenced by electronic microscope images (SEM, EDAX) and by flame atomic absorption spectrophotometry. The antimicrobial tests were performed with two fungi: *Candida albicans* and *Trichophyton interdigitale*. The obtained antimicrobial effect was considerably higher compared to the raw fabrics. Treatment of dyed fabrics with a colloidal solution based on silver chloride and titanium dioxide particles does not considerably influence colour resistance of dyes.

## 1. Introduction

**Scheme 1:**
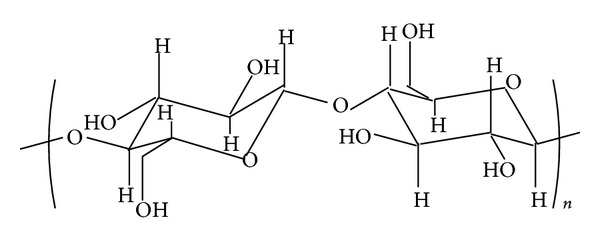
Cellulose structure.

Cotton is a common material for the production of textiles for sports, leisure, or medical uses. Itis a soft, fluffy staple fiber that grows in a boll, or protective capsule, around the seeds of cotton plants of the genus* Gossypium*. The fiber is almost pure cellulose (Scheme 1). Under natural conditions, the cotton bolls will tend to increase the dispersion of the seeds. The chemical composition of cotton is as follows: cellulose 91.00%, water 7.85%, protoplasm, pectins 0.55%, waxes, fatty substances 0.40%, and mineral salts 0.20% [1]. Cotton has excellent moisture absorption ability. However, the ability of cotton fibers to absorb large amounts of moisture makes them more prone to microbial attack under certain conditions of humidity and temperature. Cotton may act as a substrate, becoming a suitable medium for bacterial and fungal growth [2, 3]. Therefore, cotton fibers are treated with numerous chemicals to get better antibacterial cotton textiles.

In general, antimicrobial properties are chemically or physically imparted to textile materials by incorporating various functional antibacterial agents onto fibers or fabrics by different techniques [4–6]. The various strategies used to attach these agents to the fabric include the graft polymerization of N-halamine monomers onto textile substrate [7], the addition of N-halamine additives into the electrospinning dope of fibers [8], the immobilization of enzymes onto ester-crosslinked cotton fabrics [9], the placement of quaternary ammonium salt onto cotton fabrics using a covalently bound adduct [10, 11], and the attachment of chitosan to cotton fabrics via crosslinking agents [12].

Among the various antibacterial agents, silver particles have shown strong inhibitory and antibacterial activity and have a relative low toxic effect on the human body at small doses for short times [13]. These particles can be incorporated in several kinds of materials, among which clothes. These clothes with silver particles are sterile and can be used to prevent or to minimize infection with pathogenic bacteria [14]. The characteristics of silver which contribute to its scientific interest are as follows. First, yeasts, fungi, viruses, and a broad spectrum of anaerobic, aerobic, Gram-positive, and Gram-negative bacteria are susceptible to the effects of ionic silver [15–18]. Regarding mycoses, they can be considered a potential antifungal agent [1]. Recent studies revealed the effects of silver on some species of fungi, particularly* Candida* genus. However, few studies have been performed that mention effects on dermatophyte fungus genus* Trichophyton* [19–21]. Second, when used in proper concentrations, the amount of silver necessary to exhibit antimicrobial activity is, for the most part, not toxic to mammalian cells [22–24]. Third, bacteria have a relatively low propensity to develop a tolerance to silver [18].

Besides the numerous ways by which antimicrobial properties can be accomplished in textiles, mentioned above, plasma-based treatments used to create antimicrobial coatings on textile deserve special attention due to some unique properties and growing demands on the environmental friendliness of finishing processes for surface modification and coating of textiles.

**Scheme 2:**
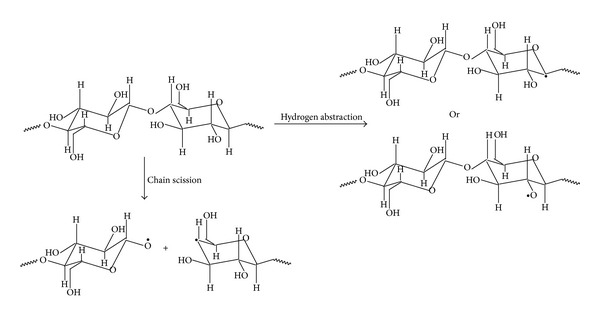
Plasma action on cellulose chains.

**Scheme 3:**
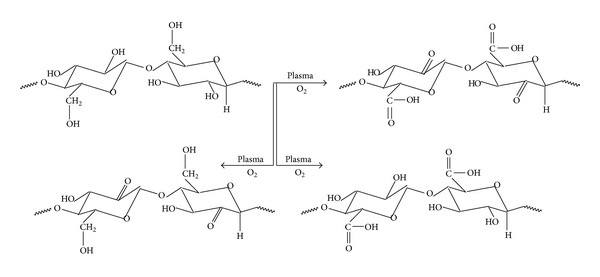
Possible reactions occurring as a result of treating cellulose with* Oxygen plasma.*

Plasma-based antimicrobial treatments of sensitive products make the subject of several research studies. There is a huge potential to use plasma for effective antimicrobial treatment [25–30]. Plasma has been defined as an ionized gas with mixture of charged particles, excited atoms or molecules, neutral particles, free radicals, and photons produced by discharges at atmospheric pressure or low pressure. Plasma treatments have been used to induce both surface modifications and bulky property enhancements of textile materials. These treatments can improve dyeing rates of polymers, develop color fastness and wash resistance, increase adhesion of coatings, and modify wettability of fibers and fabrics [25–28]. However, the most desired function of the plasma treatments is to improve the durability of the finishing rather than increase water, dyestuff, or chemical uptake [29]. The general reactions occurred by plasma treatment are the oxidation of the surface of a material, the generation of radicals, and the edging of the surface. Plasma can abstract hydrogen from the polymeric chain of cellulose or can split chains (see Scheme 2). In the case where cellulose is treated with plasma in the presence of oxygen, a surface activation can be performed by substituting hydrogen in a polymeric chain with other groups such as =O, –OH, and –COOH, according to Scheme 3. Effects of plasma on polymeric materials are similar to those produced by electron beam (ionizing radiation). Crosslinking and grafting of synthetic polymers and degradation of natural fibers mainly occur by irradiation [31–35].

**Scheme 4:**
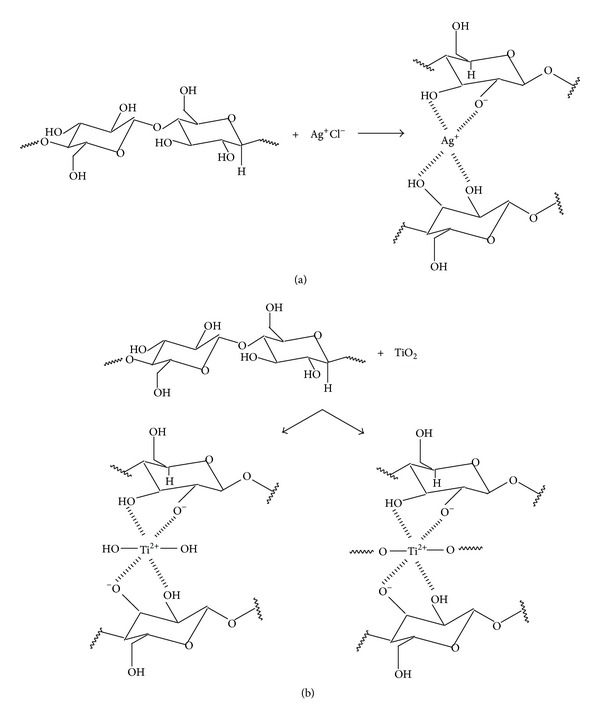
Possible reactions occurring as a result of treating cotton/cellulose with silver chloride (a) and titanium dioxide (b) in the presence of water in weak acid medium.

The objective of this research is to impart added value on cotton fabrics to improve the quality of life and thus to tap new markets with the product. For this aim, the effects of oxygen plasma treatment on cotton fabrics finished with an antibacterial chemical agent based on silver chloride and titanium dioxide particles were investigated. Silver or its compounds have been recognized for their broad spectrum antimicrobial activities. Silver inactivates bacteria by interacting with the thiol (–SH) groups of bacterial proteins and enzymes. Despite the usefulness of silver containing antimicrobial agents, bacterial resistance has developed because of uncontrolled and increasing application of these agents. To reduce the risk of emergence of silver-resistant microorganism, a second antimicrobial compound titanium dioxide has been incorporated. In the presence of ultraviolet A, water, and oxygen, titanium dioxide generates highly reactive OH radicals that are microbicidal [36].  Scheme 4 shows the possible chemical reactions that can occur as a result of treating cellulose with silver chloride and titanium dioxide in the presence of water in weak acid medium.

In this study, some of the woven fabric samples were exposed to oxygen gas plasma and then treated with antibacterial chemical agents through the padding process using a colloidal solution of silver chloride and titanium dioxide. The antibacterial activities of the washed and unwashed samples were evaluated against both* Candida albicans* and* Trichophyton interdigitale*. In addition to these, the antibacterial activities of the untreated and only oxygen plasma treated fabrics were also assessed.

## 2. Experimental

The* woven* fabrics were subjected to the following manufacturing process: weaving process, preliminary finishing of the fabrics, plasma surface cleaning, dyeing, and antimicrobial finishing.

### 2.1. The Woven Cotton Fabrics

They were manufactured in the National R&D Institute for Textile and Leather microproduction station on UNIREA and SOMET weaving machines. The fabrics were designed in order to meet the requirements of the final end products.

### 2.2. Preliminary Finishing of the Fabrics

Raw fabrics (sample I and sample II) have undergone the following stages of the preliminary finishing technological process: kier-boiling, washing, rinsing, drying, bleaching, washing, and rinsing.

### 2.3. Plasma Treatment: Cleaning and Activating the Surface for Dyestuff Affinity

The selected fabrics were subjected to oxygen plasma treatment for wettability functionality in a plasma roll-to-roll low-pressure installation fitted with 2 300 W generators, one with frequency in the MHz range and the other with frequency in the KHz range and power: 50 W. Working parameters were gas type: oxygen, pressure: 20 mTorr, temperature: 22°C, and time: 25 min (roll-to-roll).

### 2.4. Fabric Dyeing Sample I

#### 2.4.1. Blue Colour Dyeing (Sample I b)

It was done using the Optisal Royal Blue 3RL direct dye. To impregnate the dye, the concentration of Optisal Royal Blue 3RL was 1.5%, salt content was 40 g/L, temperature was 98°C, process time was 1 hour, pH was 8, and bath ratio was −1/3. The following stages were then performed: hot washing: 10 min at 60°C, soaping with Kemapol SR 40: 1 g/L, warm washing: 10 min in three stages, and rinsing: 10 min in cold water.

#### 2.4.2. Green Colour Dyeing (Sample I g) with Kemazol Green 6B Reactive Dye

To impregnate the dye, the concentration of Kemazol green 6B was 3%, salt content was 40 g/L, soda ash content was 5 g/L, caustic soda content, 38° Be was 2 mL/L, and Kemapon PC was 1 g/L. The following stages were then performed: warm washing: 10 minutes in three stages, rising: 10 min in cold water, soaping with Kemapol: 1 g/L at 95°C for 10 minutes, warm washing: 10 minutes in three stages, and rising: 10 min in cold water.

### 2.5. The Antimicrobial Finishing Used

We used silver chloride and titanium dioxide sanitized T 27-22 silver by Sanitized AG, Switzerland, with the following characteristics: pH at 20°C: 6.3, nonionogenic, density at 20°C: 1.0 gm/cm^3^, temperature stability: up to 190°C, appearance: white to light grey suspension, solubility: mixable with water, compatible with other textile chemicals, and fastness: excellent wash, dry cleaning, and ironing.

A colloidal solution of silver chloride and titanium dioxide was made in a concentration of 0.6 g sanitized T 27-22 silver/L and a pH of 6 at a temperature of 25–30°C.

Fabrics were impregnated with the obtained solution through the padding process (impregnation of a textile substrate in a bath in order to dye it or chemically treat it with a liquid or paste followed by wringing by passing the textile material through squeeze rolls to remove part of the liquid or paste from the substrate). The uptake of silver solution onto the 100% cotton fabrics was 80%. Impregnated fabrics were then dried at 130°C. The duration of the technological process of drying varies depending on the density of thread length and on the mass per area unit of fabric. Thus, for type II fabric, drying time was 5 min, and for other types of fabrics (type I), it was 3 minutes. Throughout the antimicrobial finishing process, distilled water was used to prevent any possibility of contamination or inhibition of targeted properties.

Table 1 presents samples obtained and analyzed in the paper.

### 2.6. Laboratory Tests

#### 2.6.1. Physical-Mechanical Characteristics

They were determined in accordance with standardized methods presented in Table 2.

The standard test procedure SR EN 12127/2003 was followed for determining the fabric mass and Karl Schroder instrument was used for the measurement of fabric thickness according to SR EN ISO 5084/2001. Hounsfield test equipment England was used for the measurement of breaking force and breaking elongation (according to SR EN ISO 13934-1-2004). Tear strength of flat textile materials was determined according to standard SR EN ISO 13937-3-2002 using an Elmendorf pendulum device.

Tests were carried out in a standard conditioning and testing atmosphere according to SR EN ISO 139:2005, at a temperature of (20 ± 2)°C and a relative humidity of (65.0 ± 4)%. The samples tested are knitted fabrics and woven fabrics made of one, two, and three textile material layers with various fibrous compositions.

#### 2.6.2. Surface Morphology

Surface morphology of textile material was observed using scanning electron microscope (SEM) type Quanta 200-FEI, Olanda, GSED detector, HV = 15 kV accelerating voltage, spot 4.5. Mode is low vacuum (pressure inside the 200P). This device enables qualitative and quantitative microanalysis by energy-dispersive X-ray spectrometry and qualitative and quantitative analysis of chemical elements (EDAX). The device is fitted with a SDD Apollo 10 detector.

#### 2.6.3. Heavy Metal Content

It was determined by flame atomic absorption spectrophotometry using an atomic absorption spectrometer/graphite furnace, flame, VGA (AAS 800, Varian, Australia), according to SR ISO 6486-1/1997, SR ISO 8288/2001. These methods highlight the existence of the following metals: silver, chromium, cobalt, nickel, copper, cadmium, and lead.

#### 2.6.4. Microbiological Tests

They were performed according to ISO 20743:2007 through the absorption method, test method where the microbial suspension is inoculated directly on the samples. The surface of tested materials was approximately 1.5 cm^2^. The microbial suspension was inoculated directly on the material, in previously sterilized test tubes, which were subsequently sealed with parafilm and incubated for 24 hours, at temperatures of 37°C for* Candida albicans* and 29°C for* Trichophyton interdigitale*. After 24 hours, cells were recovered from test tubes with saline solution, and the recovered inoculum was inoculated on a Sabouraud Agar nutritive medium for* Candida albicans* and Czapek-Dox for* Trichophyton interdigitale*. After incubation, the number of viable cells was counted and the microbial reduction degree was calculated depending on the initial number of colony-forming units on untreated textile materials (control). The initial concentration of tested inoculum was 2.4 × 10^4^ UFC/mL for* Candida albicans* and 1.1 × 10^4^ UFC/mL for* Trichophyton interdigitale.*

To observe microbiological samples (fungi cultures) the Discovery V8 Stereomicroscope was used.

#### 2.6.5. Coloristic Resistance of Dyed Fabrics

To determine dye resistance of treated fabrics, for each sample, the following were analyzed*: *dye resistance to washing (according to SR EN ISO 105-C06:2011), dye resistance to perspiration (according to SR EN ISO 105-E04:2013), dye resistance to rubbing (according to SR EN ISO 105-X12:2003). and dye resistance to water (according to SR EN ISO 105-E01:2003).

#### 2.6.6. Colour Difference

In the case of dyed fabrics, it was determined according to SR EN ISO 105 J 03: 2010. Colour difference is constituted of three components containing differences between control samples and test samples. These components are DL*, DC*, and DH*.

DL* is the brightness component weighted with the brightness tolerance (Δ*L*/IS_*L*_). This component is represented as Δ*L*_cmc_. If Δ*L*_cmc_ is positive, the tested sample is brighter than the control sample. If Δ*L*_cmc_ is negative, the tested sample is darker than the control sample.

DC* is the saturation component weighted with the chromatic tolerance (Δ*C*_ab_/cS_*c*_). This component is represented as Δ*C*_cmc_. If the value of Δ*C*_cmc_ is positive, the tested sample is more saturated than the control sample. If the value of Δ*C*_cmc_ is negative, the tested sample is less saturated than the control sample.

DH* is the hue component weighted with the tolerance (Δ*H*_ab_/S_*H*_)  . This component is represented as Δ*H*_cmc_. If Δ*H*_cmc_ is positive, the hue difference of the specimen is anticlockwise from the reference in the CIELAB a*, b* diagram. If Δ*H*_cmc_ is negative, the hue difference of the specimen is clockwise from the reference in the CIELAB a*, b* diagram.

## 3. Results and Discussions

### 3.1. Physical-Mechanical Properties of the Fabrics

The physical-mechanical properties of the raw fabrics and fabrics which were subjected to oxygen plasma treatment are evidenced in Table 2. The presented data show that oxygen plasma treatment leads to a slight decrease in breaking force, breaking elongation, tear resistance, abrasion resistance, water vapor permeability, and air permeability. These changes of physical-mechanical characteristics may occur as a result of treating cotton fabrics with* oxygen plasma,* when oxidation of the material surface, the generation of radicals, polymer chain scission, and so forth may occur (see Schemes 2 and 3). At the same time, fabric width and fabric thickness change, mass/surface, fabric density increases slightly, and, as a result, thermal resistance and water vapor resistance improve slightly.

### 3.2. SEM and EDAX Analysis

**Figure 1:**
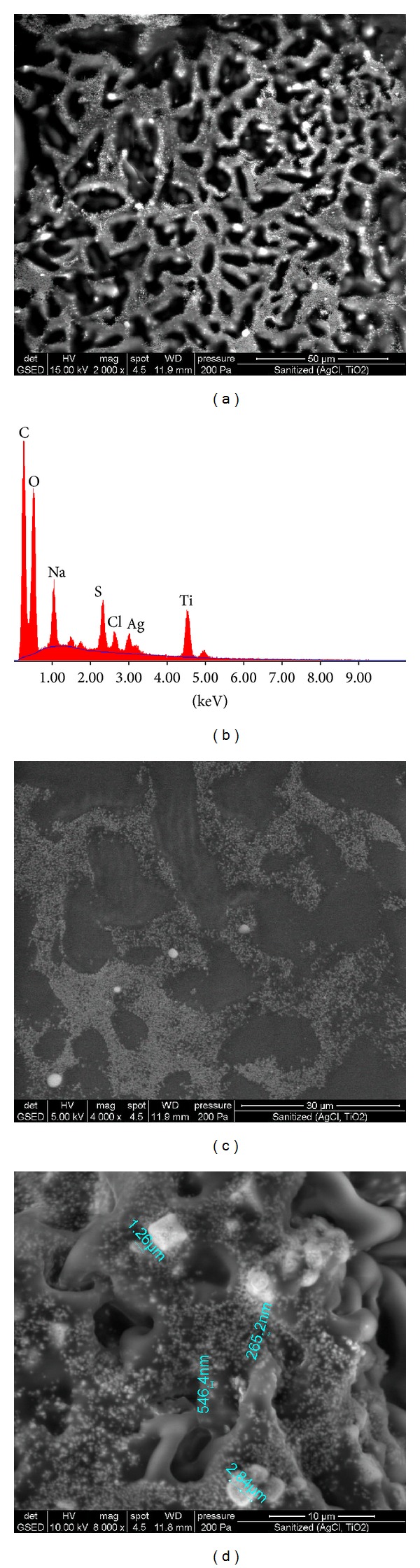
SEM and EDAX images for colloidal solution of silver chloride and titanium dioxide sanitized T 27-22 silver: (a) SEM image for colloidal solution at resolution ×2000, (b) EDAX image for colloidal solution, (c) SEM image for colloidal solution at resolution ×4000, and (d) SEM image for colloidal solution at resolution ×8000.

**Figure 2:**
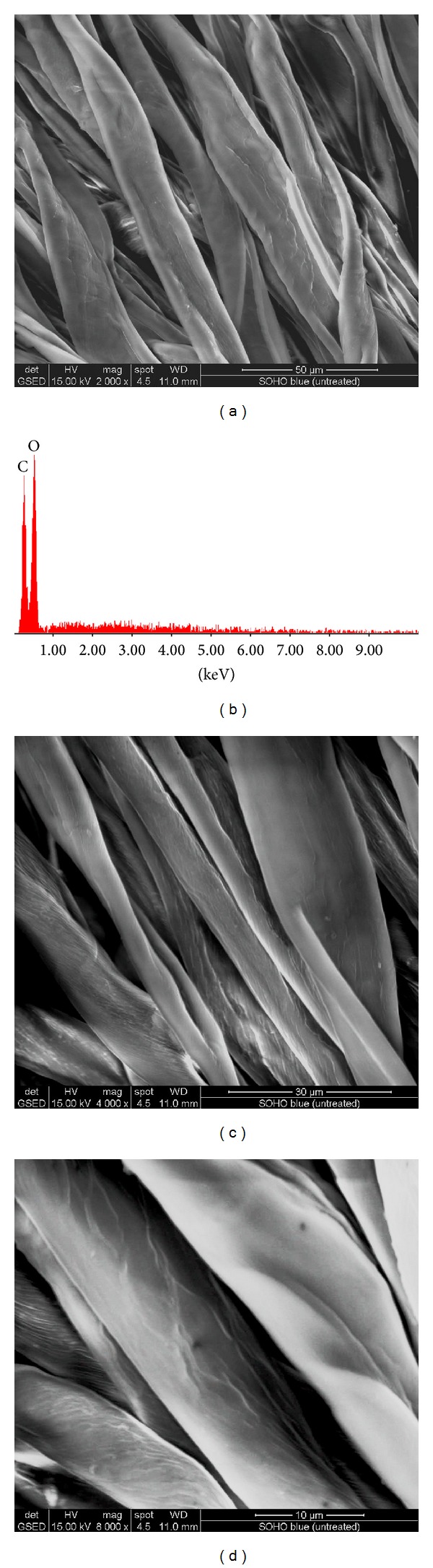
SEM and EDAX images for sample Ib (control) (plasma functionalized type I bleu cotton 100%): (a) SEM image for sample Ib at resolution ×2000, (b) EDAX image for sample Ib, (c) SEM image for sample Ib at resolution ×4000, and (d) SEM image for sample Ib at resolution ×8000.

**Figure 3:**
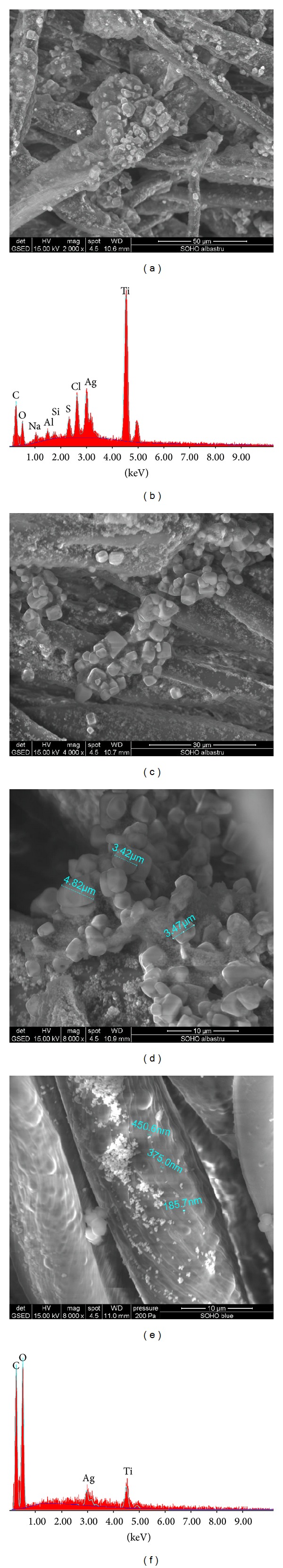
SEM and EDAX images for sample Ib s (antimicrobial finished plasma functionalized type I bleu cotton 100%): (a) SEM image for sample Ibs at resolution ×2000, (b) EDAX image for sample Ibs, (c) SEM image for sample Ib at resolution ×4000, (d) SEM image for sample Ibs at resolution ×8000, (e) SEM image for sample Ibs after 5 washes at resolution ×8000, and (f) EDAX image for sample Ibs after 5 washes.

**Figure 4:**
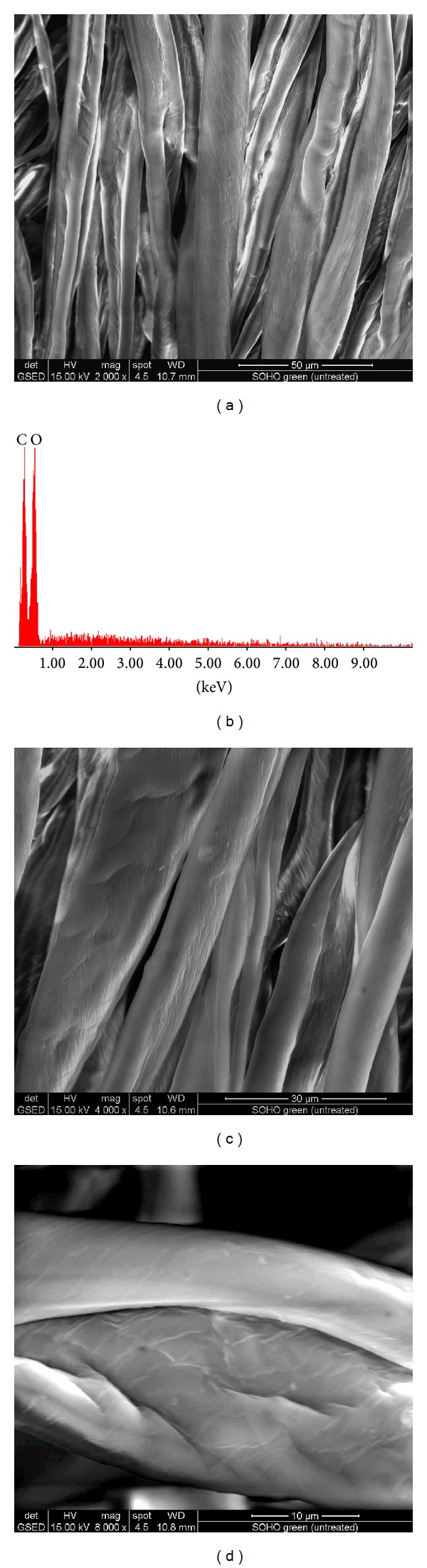
SEM and EDAX images for sample Ig (control) (plasma functionalized type I green cotton 100%): (a) SEM image for sample Ig at resolution ×2000, (b) EDAX image for sample Ig, (c) SEM image for sample Ig at resolution ×4000, and (d) SEM image for sample Ig at resolution ×8000.

**Figure 5:**
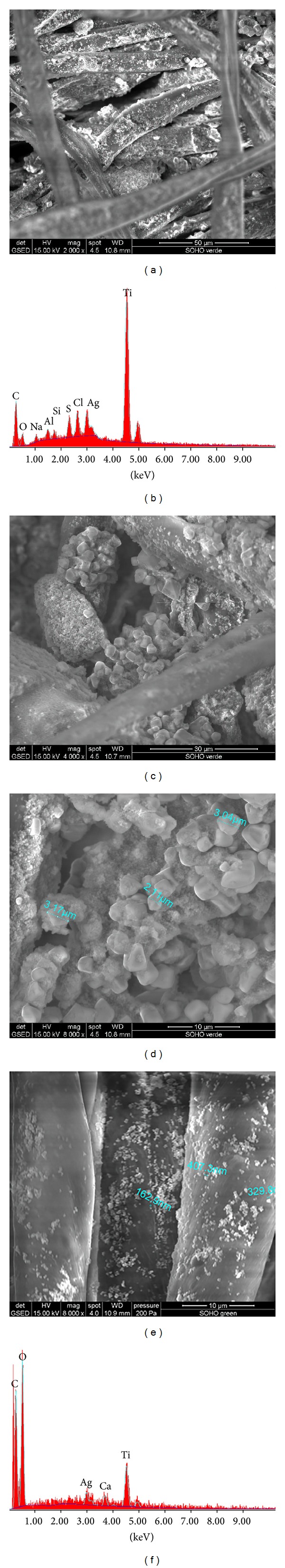
SEM and EDAX images for sample Igs (antimicrobial finished plasma functionalized type I green cotton 100%): (a) SEM image for sample Igs at resolution ×2000, (b) EDAX image for sample Igs, (c) SEM image for sample Ig at resolution ×4000, (d) SEM image for sample Igs at resolution ×8000, (e) SEM image for sample Igs after 5 washes at resolution ×8000, and (f) EDAX image for sample Igs after 5 washes.

**Figure 6:**
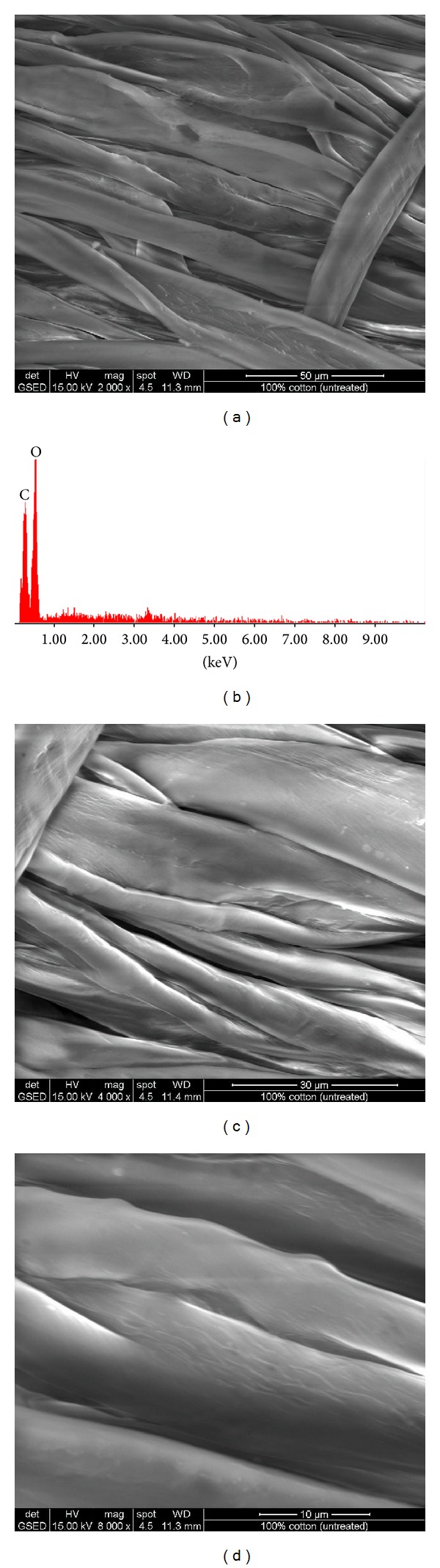
SEM and EDAX images for sample II (control) (plasma functionalized type II cotton 100%): (a) SEM image for sample II at resolution ×2000, (b) EDAX image for sample II, (c) SEM image for sample II at resolution ×4000, and (d) SEM image for sample II at resolution ×8000.

**Figure 7:**
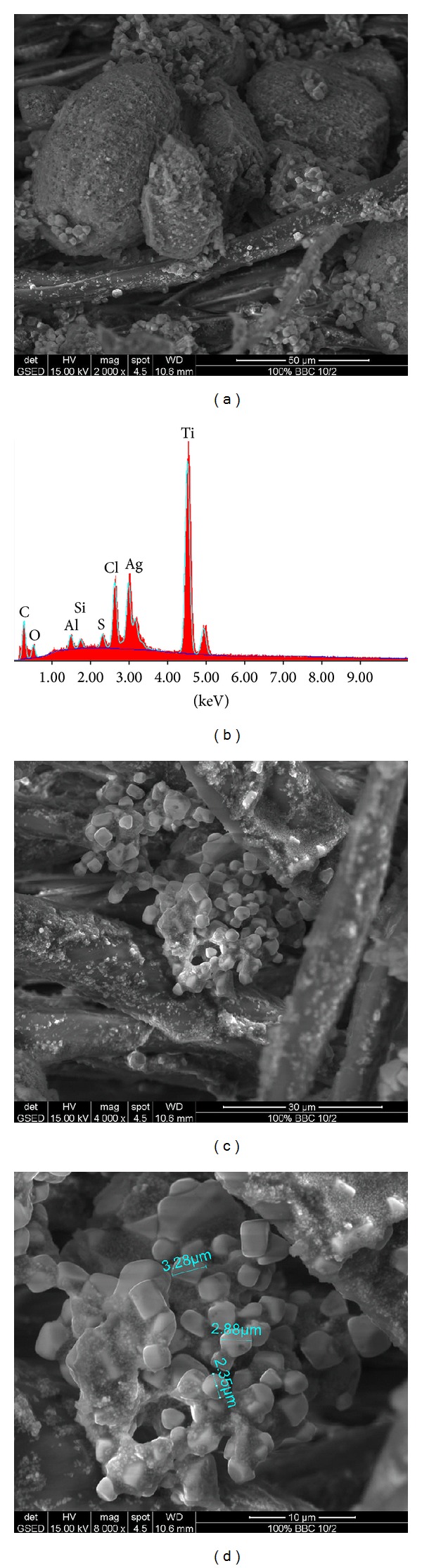
SEM and EDAX images for sample IIs (antimicrobial finished plasma functionalized type II cotton): (a) SEM image for sample IIs at resolution ×2000, (b) EDAX image for sample IIs, (c) SEM image for sample IIs at resolution ×4000, and (d) SEM image for sample IIs at resolution ×8000.

SEM and EDAX images of treated fabrics are presented in Figures 1, 2, 3, 4, 5, 6, and 7 for colloidal solution of silver chloride and titanium dioxide sanitized T 27-22 silver, control sample and for sample antimicrobial finished plasma functionalized.

SEM and EDAX images of colloidal solution of silver chloride and titanium dioxide sanitized T 27-22 silver are presented in Figure 1. SEM images of the solution show the existence of  larger clusters of particles, and particle sizes range between 100 and 1000 nm. EDAX analysis shows, along with Ag, Cl, Ti, and O from AgCl and TiO_2_, the presence of elements such as C, Na, and S, which may be due to the stabilizer used for this colloidal solution.

SEM images of control samples and antimicrobial treated samples indicate that treated fabrics were charged. From the SEM analysis of sample antimicrobial finished, white clusters of particles with an approximate size of 2–5 *μ*m were observed (Figures 3(d), 5(d), and 7(d)) and were responsible for high peaks of silver and titan (Figures 3(b), 5(b), and 7(b)). Other chemical elements present in the spectrum may be due to the dust deposited on the fabric, respectively, to the stabilizer used for the colloidal solution (e.g., Na, S, and Si). Treatment on the fabric surface shows uneven arrangement microparticles attached onto the fibers (Figures 3, 5, and 7).

After 5 washing cycles as per ISO 6330, it was noticed that the treatment persisted on the fabrics, but the size of particle clusters shrank (Figures 3(e) and 5(e)) and the amount of Ag and Ti was reduced (Figures 3(f) and 5(f)). For this reason, the new antimicrobial cotton fabrics may be used in several medical applications, including wound dressings, prosthetic valves, hospital uniforms, and linens [37].

### 3.3. Determination of Silver Content on Antimicrobial Finished Fabrics by Flame Atomic Absorption Spectrophotometry

The results obtained, presented in Table 3, have highlighted the existence of silver on the antimicrobial finished fabrics. The other heavy metals (chromium, cobalt, nickel, copper, cadmium, and lead) which may be determined using this method have been undetectable.

### 3.4. Microbiological Tests on* Candida albicans* and* Trichophyton interdigitale*

The antimicrobial tests were performed with two fungi*: Candida albicans *and* Trichophyton interdigitale.*

#### 3.4.1. Microbiological Tests on* Candida albicans*

*Candida albicans* is a diploid fungus that grows both as yeast and filamentous cells and a causal agent of opportunistic oral and genital infections in humans [38, 39], and candidal onychomycosis, an infection of the nail plate. At the same time, it may infect the skin, mucous membranes, nails, and gastrointestinal tract. The incidence of candidal infection is rising due to the growing number of individuals with suppressed immune function caused by malignancy, HIV infection, antibiotic use, steroid use, or chemotherapy [38, 39]. In addition, common health problems, including diabetes mellitus and obesity, can also predispose an individual to candidal skin infection [38, 39].* Candida albicans* is a major cause of nosocomial infections (infections acquired during medical care); contaminated health care workers and biomaterials are common sources of these infections [40]. For example,* Candida albicans* is the most common fungus isolated from surgical wounds; Giandoni et al. demonstrated that asymptomatic candidal infection may delay wound healing [41, 42]. In addition,* Candida albicans* is the most commonly isolated fungal species in intensive care unit (ICU) patients; candidal infection is associated with ICU patient mortality [43]. It is interesting to note that* Candida albicans* may also cause enzymatic degradation of common textile dyes [44]. For example, Vitor and Corso demonstrated degradation of Direct Violet 51 azo dye by* C. albicans*, which resulted in removal of color [44].

**Figure 8:**
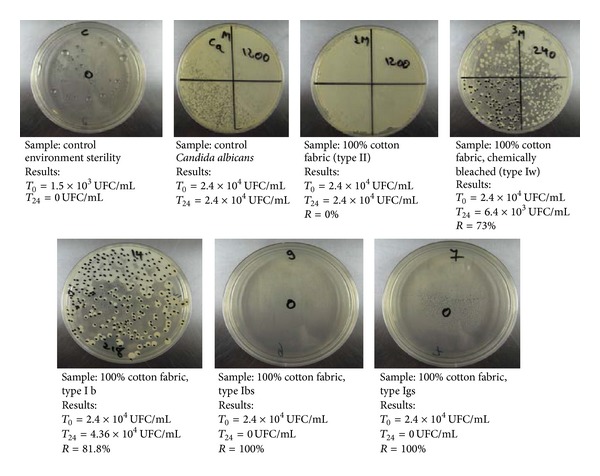
The result of microbiological tests on* Candida albicans* (according to ISO 20743:2007, absorption method).

As a result of microbiological tests (see Figure 8) on* Candida albicans*, materials functionalized in oxygen plasma and then antimicrobially treatedexhibited excellent results after testing on* Candida albicans*, the 2 treated materials (Igs and Ibs) having a microbial reduction rate of 100%. These were compared with control materials (untreated control samples) starting from a microbial concentration in the tested inoculum of 2.4 × 10^4^ UFC/mL. Untreated control materials (control samples) have not shown microbial reduction. However, microbial reduction was noticed in the case of textiles untreated with dedicated antimicrobial agents. Thus, in the case of fabric type I, chemically bleached (Iw), a microbial reduction of 73% was noticed. This can be due to chemical reagents used in the beaching process (peroxides, detergents, quaternary ammonium compounds, etc.).

#### 3.4.2. Microbiological Tests on* Trichophyton interdigitale*

*Trichophyton interdigitale* is a species of* Trichophyton* [45]. It can produce penicillin. Some sources equate it with “*Trichophyton mentagrophytes*” [46]. As such, it is one of the three common fungi which cause ringworm in companion animals. It is also the second most commonly isolated fungus causing so-called tinea infections in humans, and the most common or one of the most common fungi that causes zoonotic skin disease (i.e., transmission of mycotic skin disease from humans to animals, and the reverse). The fungus has a major natural reservoir in rodents but can also infect pet rabbits, dogs, and horses [46].

**Figure 9:**
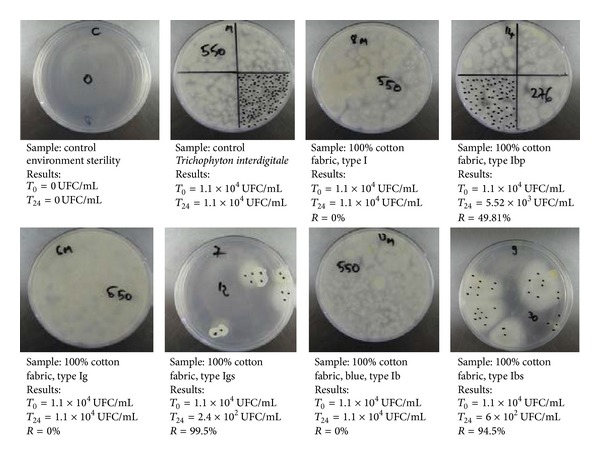
The result of microbiological tests on* Trichophyton interdigitale *(according to ISO 20743:2007, absorption method).

As a result of microbiological tests (see Figure 9) on* Trichophyton interdigitale*, antimicrobially treated materials exhibited very good results,Igsfabric with a reduction rate of  99.5% and Ibsfabric with a reduction rate of 94.5%. These were compared with untreated control materials, starting from a microbial concentration in the tested inoculum of 1.1 × 10^4^ UFC/mL.

A microbial reduction of 49.81% was noticed for the Ibp fabric, possibly due to the formation of reactive chemical species which lead to chemical reactivity phenomena with the biological material.

Given the complex nature of antimicrobial testing and the varied nature of tested antimicrobial agents, there is a series of factors that may considerably influence test results: (a) mechanical retention of microbial cells on textiles, depending on the surface morphology, ultimately leading to complete recovery of cells on the surface of textile materials; (b) the dispersion degree of the antimicrobial material on the material surface, in low agent concentrations avoiding agglomeration in coarse particles, thus reducing contact surface of the agent with the microbial cells; (c) the presence of pretreatment compounds from bleaching and dyeing processes; (d) a variation of the hydrophobic/hydrophilic nature of textiles which may influence the contact degree of the microbial inoculum with the textiles.

### 3.5. Resistance of Dyes for Antimicrobially Treated Fabrics

The experimental study and analysis of values obtained for colour resistance highlight the following behaviour: (1) antimicrobial treatment slightly influences colour change. Release on multifibres is comparative in both dyeing situations, and the silver deposition does not change values of marks obtained compared to control samples; (2) marks for dye resistance to dry and wet abrasion are also almost identical in comparative situations; (3) dye resistance to water is not influenced by the antimicrobial treatment, and the obtained marks do not change in any comparative situation; (4) dye resistance after 90 hour exposure to artificial light is not influenced by silver and titanium ion deposition on dyed fabrics in the two colours, blue and green, and compared to fabrics not dyed, values of evaluation marks both on the grayscale and on the bluescale are the same. In conclusion, treating dyed fabrics with a colloidal solution based on AgCl and TiO_2_ does not significantly influence colour resistance of dyes.

### 3.6. Colour Difference in the Case of Antimicrobially Treated Fabrics

After treating type I and type II white/not dyed fabrics with the silver ion-containing solution, the following behaviour is highlighted in both cases: (1) DL* values are negative, which means that samples treated with AgCl and TiO_2_ solution are darker than the corresponding control samples; (2) values obtained for DC* are positive, which means that samples treated with AgCl and TiO_2_ solution are more saturated than corresponding control samples; (3) as far as values obtained for DH* are concerned, for antimicrobially treated 100% cotton samples, type I and type II,they arenegative in comparison to control samples, which means that the hue difference of the sample treated with AgCl and TiO_2_ solution is in the clockwise direction compared to control samples on the CIELAB, a*, b* diagram.

After treating type I fabric dyed in the two blue and green variants with the colloidal AgCl and TiO_2_ solution, the following behaviour is highlighted: (1) DL* value is negative for the green dyed sample treated with AgCl and TiO_2_ solution, which means that it is darker than the control sample. DL* value is positive for the blue dyed sample treated with AgCl and TiO_2_ solution, which means that it is brighter than the control sample; (2) DC* values are negativein both dyeing situations, which means that dyed samples treated with AgCl and TiO_2_ solution are less saturated than control samples, (3) DH* values are negativein both dyeing situations, which means that the hue difference of dyed samples treated with AgCl and TiO_2_ solution is in the clockwise direction compared to control samples on the CIELAB, a*, b* diagram.

In conclusion, the influence of treatment with AgCl and TiO_2_ colloidal solution on dyeing colour determines a brighter or darker hue compared to the control sample, depending on the positive or negative values of DL*, more or less saturated, and depending on the positive or negative values of DC*.

## 4. Conclusion

The woven fabrics were cleaned in oxygen plasma and treated by padding with silver chloride and titanium dioxide particles for the improvement of the antimicrobial character. 

The existence of silver on the antimicrobial finished fabrics was evidenced by flame atomic absorption spectrophotometry and SEM-EDAX.

As a result of microbiological tests on* Candida albicans*, materials functionalized in oxygen plasma and then antimicrobially treatedexhibited excellent results, a microbial reduction rate of 100%. Untreated control materials have not shown microbial reduction. In the case of fabric type I, chemically bleached, a microbial reduction of 73% was noticed. This can be due to chemical reagents used in the beaching process.

As a result of microbiological tests on* Trichophyton interdigitale*, antimicrobially treated materials exhibited very good results,Igsfabric with a reduction rate of 99.5% and Ibsfabric with a reduction rate of 94.5%. These were compared with untreated control materials. A microbial reduction of 49.81% was noticed for the Ibp fabric, possibly due to the formation of reactive chemical species which lead to chemical reactivity phenomena with the biological material.

At treating dyed fabrics with a colloidal solution based on AgCl and TiO2, does not significantly influence colour resistance of dyes. The influence of treatment with AgCl and TiO_2_ colloidal solution on dyeing colour determines a brighter or darker hue compared to the control sample, depending on the positive or negative values of DL*, more or less saturated, and depending on the positive or negative values of DC*.

## Figures and Tables

**Table 1:** 100% cotton fabric samples obtained and analyzed in the paper.

Number	Sample description	Sample symbol
1	100% cotton fabric type I (control)	I
2	100% cotton fabric type I chemically bleached (control)	I w
3	100% cotton fabric type I chemically bleached antimicrobial finishing	I ws
4	100% cotton fabric type I, green (control)	I g
5	100% cotton fabric type I, green antimicrobial finishing	I gs
6	100% cotton fabric type I, blue (control)	I b
7	100% cotton fabric type I, blue, plasma treatment	I bp
8	100% cotton fabric type I, blue antimicrobial finishing	I bs
9	100% cotton fabric type II (control)	II
10	100% cotton fabric type I antimicrobial finishing	II s

**Table 2:** Physical-mechanical properties of the fabrics.

Test type	Sample I raw	Sample I plasma	Sample II raw	Sample II plasma	Standard
Fibrous composition	100% cotton	100% cotton	100% cotton	100% cotton	ISO 1833:1995

Mass/surface, g m^−2^	189	191	442	447	EN 12127:2003

Fabric width, cm	154.7	153	152	150	SR EN 1773:2002

Fabric weave	Plain weave	Plain weave	Plain weave	Plain weave	SR 6431:2012

Fabric thickness, mm	0.66	0.70	1.19	1.20	SR EN ISO 5084/2001

Fabric density number of yarns/10 cm					SR EN 1049/2:2000
Warp	180	192	108	109	
Weft	160	176	85	86	

Air permeability, lm^−2^s^−1^; 100 Pa	555.9	497.3	159.4	154.3	SR EN ISO 9237/1999

Water vapor permeability, %	34.7	32.2	31.3	30.1	SR 9005-1979

Abrasion resistance, abrasion cycles	14813	13900	20100	20050	SR EN ISO 12947-2:2002

Thermal resistance (*R*_ct_), m^2^ K W^−1^	0.0023	0.0173	0.03072	0.03175	SR EN 31092 ISO 11092/1997

Water vapor resistance (*R*_et_), m^2^ Pa W^−1^	5.502	5.660	7.734	7.983	SR EN 31092 ISO 11092/1997

Breaking force, N					SR EN ISO 13934-1-2004
Warp	504	490	1126	1100	
Weft	409	401	495.8	490	

Breaking elongation, %					SR EN ISO 13934-1-2004
Warp	26.5	25.7	25.98	17.95	
Weft	14.34	13.9	9.22	9.58	

Tear resistance, N					SR EN ISO 13937-3-2002
Warp	20.5	19.4	120.2	119.9	
Weft	21.5	20.9	49.1	46.23	

**Table 3:** Heavy metal content of samples with antimicrobial treatment.

Sample name	Analysed metal	Metal content, mg/L
Fabric type I bs	Cu, Cr, Cd, Co, Ni, Pb, and Ag	Undetectable, 0.340
Fabric type I gs	Cu, Cr, Cd, Co, Ni, Pb, and Ag	Undetectable, 0.330
Fabric type II s	Cu, Cr, Cd, Co, Ni, Pb, and Ag	Undetectable, 0.513
